# Observations on the Nature and Treatment of Beriberi

**Published:** 1828-03

**Authors:** William Hamilton

**Affiliations:** Royal Navy.


### BERIBERI.

### *Observations on the Nature and Treatment of Beriberi.*

By William Hamilton, Esq. Royal Navy.

By the different authors who have touched upon the subject, from the days of Bontius (who first described the disease, and gave it a place in medical nomenclature,) down to those of Dr. Christie, and from his time to the present, Beriberi has been considered as a disease of debility, in the cure of which any thing approaching to a depletory mode of treatment has been most religiously avoided. Finding, from a medical friend lately returned from India, that little or no deviation from the accustomed practice so long pursued in such cases had taken place at the time he quitted that country, in June 1825,—a short time previously to which beriberi had proved more than usually fatal, particularly in the northern division of the Madras settlement,—I have, at the request of the individual alluded to, been induced to communicate, through your Journal, the following observations on the nature and treatment of this complaint, as tending to favor the propriety of a very opposite mode of practice to that so generally had recourse to for its cure. My object being chiefly to confine myself to facts, and by a simple statement of such lead the attention of others to the subject, I shall, in the course of my remarks, avoid, in as far as possible, touching upon such points as would naturally involve either hypothesis or theory.

This very singular endemic (occurring in particular parts of the East Indies, and which, I believe, has not been described as having existed in any other quarter of the world,) appears to be principally, I may say almost entirely, confined to the Island of Ceylon, the Malabar coast, and that tract of country reaching from Madras as far north as Ganjam, and in no part extending inland more than forty miles, forming what is called the northern division of the Madras settlement. It is most prevalent during the decline of one monsoon and setting-in of the other, when the atmosphere is completely loaded with cold, raw, damp vapours, and the vicissitudes of temperature are greater than at any other period of the year; and though in a less degree does a residence in the neighbourhood of the coast seem essential for its production, than a stay for some months on a station where it prevails, yet the instances are comparatively rare where it has been found to occur at a distance from the sea exceeding sixty or seventy miles.

Individuals of very different constitutions and habits of body seem liable to be affected by it; but such as lead a sedentary and debauched life, and who are much exposed to vicissitudes of weather and sudden changes of temperature, are unquestionably those most subject to its attacks; and an individual once having suffered from it appears more liable to a future attack than one who had never been the subject of it.

I have only had an opportunity of seeing the disease under two forms: viz. that in which the symptoms were at first mild, and gradually increased in severity; and that in which the symptoms were even from the first rather urgent, increased more rapidly, and, unless speedily relieved, soon led to the most alarming appearances, and finally proved fatal. There is another variety mentioned by Dr. Christie, which that author considers equally fatal, and in which "there was not any swelling observable externally, but the patient, with the other symptoms, had evidently the bloated leucophlegmatic face of a dropsical person." Dr. Rogers, of the Hon. East India Company's Madras establishment, likewise mentions this variety of the complaint, in a Thesis written by him, and printed at Edinburgh in the year 1808. "Hydrops asthmaticus," says he, "tumore nullo externo comitante, nobis ante oculos interdum versatus fuit; his autem in exemplis ægro exitium semper attulit; vultusque et tumidus et leucophlegmaticus, hydrope laborantis sane proprius ægrotanti fuit." This form of the complaint I have never seen. The first case that fell under my care was one in which the disease appeared under its milder form. There was from the commencement slight dyspnœa, with stiffness of the lower extremities; pulse quick and sharp; skin dry and rough. Supposing it only a slight feverish attack, (the individual having been exposed the preceding night to the damp and cold winds which prevail during the change of monsoon,) I prescribed a solution of emetic tartar, to be given in small repeated doses, for the purpose of acting on the stomach, bowels, and skin, and directed that he should be kept warm; having very little doubt that, by next morning, I should find my patient much relieved. To my great mortification, however, I found all the symptoms much aggravated, particularly the stiffness he complained of in his legs and thighs, which now amounted to an almost complete paralysis. From this time there was no mistaking the nature of the complaint, and, though I strictly followed the plan of treatment recommended by Dr. Christie, and by him found so successful, the poor fellow died on the evening of the tenth day from the commencement of the attack, having for some days previously laboured under all the symptoms generally present in beriberi. This body, from particular circumstances, I was prevented examining.

In the second case which I had an opportunity of attending, the disease, from the time that I first saw it, appeared under its more aggravated form. The symptoms were as follow:—Great debility, with difficulty of respiration; a sense of weight and oppression at the lower end of the sternum; and an almost paralytic state of the thighs and legs, which, soon after the commencement of the attack, became œdematous, as did also the face, and indeed the greater part of the body; with a general sensation of coldness over its surface; pulse 120, small, feeble, and intermitting. All these symptoms went on increasing until the death of the patient, which took place within forty-eight hours from the time he was first attacked. A short time previously to his death, he was seized with a violent fit of vomiting, spasms of the abdominal muscles, and increased dyspnœa, which carried him off.

Though the plan of treatment recommended by Dr. Christie had in the first case proved unsuccessful, such was my confidence in that author, from the very decided manner in which he speaks of his success, that I was a second time induced to follow his footsteps in the treatment of this complaint; but, I am sorry to say, as unsuccessfully as before. I now determined to examine the body, and, if possible, discover some indication or other by which I might be guided in my future treatment of the complaint, should a case of the kind again occur to me. I shall here state the particulars of this examination, together with the mode of treatment I was afterwards led to adopt, by which I succeeded in completely curing my next three patients; in two of whom the symptoms were, in the first instance, still more alarming than in either of the cases which terminated fatally. Leaving the country soon afterwards, I was deprived of an opportunity of giving it a more extensive trial.

I carefully examined the contents of the cranium, thorax, and abdomen. Upon removing the skull-cap, I found upwards of an ounce of serum effused between the dura mater and tunica arachnoidea; and in two or three different places there appeared dark red-coloured patches, one of which was exceedingly vascular, and extended into the substance of the brain, to the depth of from a quarter to half an inch. There was likewise found considerable effusion in all of the ventricles except the fifth, or that cavity formed by the separation of the laminæ of the septum lucidum. In the base of the cranium, upon the brain being removed, there appeared upwards of four ounces of fluid tinged with blood. The lungs were very much loaded with dark-coloured blood; and in both cavities of the thorax there was found extensive effusion. The heart was of a pretty healthy appearance; nor did the pericardium seem to contain a much greater quantity of fluid than that generally found in it; both on its external surface, however, and likewise internally, there existed very evident marks of inflammation.

The diaphragm, particularly towards the right side, appeared considerably inflamed. The stomach was of a healthy appearance, and contained about six ounces of a dark brown-coloured liquid. The liver was very evidently larger than natural, and appeared even still more loaded than the lungs: on cutting through its substance, the blood, from different points, trickled out almost in a continued stream; and indeed all, even the most minute, vessels seemed completely gorged; as were likewise those of the mesentery and pancreas. In several places on the surface of the intestines there appeared a sort of efflorescence, but, upon the whole, they presented nothing very remarkable. From three to four pounds of fluid were found in the cavity of the abdomen; and in the cellular texture, almost all over the body, there existed very extensive effusion. On examining the spinal marrow, there was found to exist the same evident marks of congestion as in the liver, lungs, &c. almost along the whole of its course, though that more particularly in the dorsal region. No other deviation from the natural healthy appearance of the parts was found to exist.

Dr. Christie mentions, that in many of the subjects he examined who had fallen victims to this disease, there was a remarkable obesity, even after a long continuance of it, and the use of mercury, antimony, and other powerful medicines. This I found to be the case in the individual whose body I examined; but he was naturally of a full habit of body, and in him the disease had not existed long.

Having fully satisfied myself, from the post-mortem appearances, that the dropsical affection and other symptoms in the case of the individual whose body I had an opportunity of examining, (and I have little doubt in every other where the disease occurs,) arose from obstructed circulation, in consequence of congestion in the internal organs; and that beriberi consequently could not be considered a mere disease of debility as described by the celebrated Bontius, and more recently by Dr. Christie, Mr. Hunter, Mr. Colhoun, and others, but a disease in the treatment of which depletion might be had recourse to with the greatest possible advantage, I fully determined, should an opportunity again present itself, (regardless of the apparent debility of the patient on the one hand, or of the doctrines of the humoral pathology on the other,) to try the effects of blood-letting, for the purpose of assisting other means in exciting reaction, determining towards the surface, and restoring the balance of circulation. For this end, in the following case, thirty ounces of blood were, upon my first seeing the patient, taken from the arm *pleno rivo;* which, from the immediate relief it afforded, I was induced to repeat: and accordingly, about twelve hours from the first bleeding, the dyspnœa and other symptoms, though much relieved, still continuing troublesome, twenty-five ounces more were drawn off in a full stream, which was followed by the same evident good effects as on the former occasion. I felt inclined even a third time to repeat the bleeding, and certainly would, had the individual been of a full habit of body, or had the disease occurred to me in a climate where debility and general relaxation were less liable to follow free depletion, and where a practitioner might, with comparative safety, carry the use of the lancet to a greater height than he would be warranted doing in a climate such as that of India.

Having resolved not to push venesection further, I had immediate recourse to mercury, upon which I now determined principally to rely in the cure of the disease, having every hope of saving my patient, provided I could succeed in bringing him speedily and effectually under its influence. Its well known effects as a great, universal, and permanent vascular stimulus, as an equaliser of the circulation, and as taking off determination from particular parts, —its diuretic effects when combined with diuretics, and diaphoretic when combined with diaphoretics, render it a medicine peculiarly well adapted for the cure of beriberi.

Soon after the second bleeding, I directed twenty grains of calomel, with thirty drops of laudanum, to be given, and had the patient laid upon the frame of a common sea-cot, having an open ratan bottom, under each end of which was placed a block of wood, for the purpose of raising it a certain distance from the ground. A blanket, having a hole cut in it sufficiently large for allowing the head to protrude, was now thrown over all, and brought close down to the ground at the ends and on each side. Two crucibles, containing ignited charcoal, were then placed under it; by means of which the whole surface of the body was freely exposed to the fumes of the Hydrargyri Oxydum Cinerium, some of which was from time to time thrown into the crucibles. This I continued for upwards of half an hour, when, the patient feeling faint, the crucibles were removed, and the body enveloped with the blanket which covered it during the fumigation.

An hour and ten minutes having elapsed from the time the calomel was given, the dose was now repeated, with the same quantity of laudanum as before: soon after which he fell into a sound sleep, and continued so for upwards of three hours. Upon his awaking, I found him in a state of copious perspiration, the pulse increased in strength, and the dyspnœa not near so troublesome. I now again repeated the calomel, with six grains of gamboge, for the purpose of acting on his bowels, and omitted the laudanum; had also the crucibles replaced, and the body again exposed to the fumes of the Hydrargyri Oxydum Cinerium; which, from this time, together with scruple doses of calomel, and friction over the surface of the abdomen and thighs with the Unguentum Hydrargyri Fortius and Liquor Ammoniæ, was repeated from every three to four hours until ptyalism was fully established, (which, notwithstanding the very active means had recourse to, required from forty to forty-eight hours to effect;) after which every unfavorable symptom began speedily to disappear, and the patient's principal complaint was the soreness of his mouth.

The particulars of this case, which are nearly similar to that of two others which fell under my care, and in which the same mode of treatment proved equally successful, I beg leave to subjoin, as copied from my notes taken at the time.

I may here add that, in one of the cases, there was from the first violent vomiting, which soon, however, yielded to large and repeated doses of calomel and laudanum, together with the application of strong mustard sinapisms, at from 150° to 160° of Fahrenheit, to the region of the stomach; which I have likewise found singularly successful in speedily allaying the violent gastric irritability, in cases of bilious remittent fever and cholera, where the calomel and laudanum alone had completely failed of success. The individual being of a rather full habit of body, and the pulse, after the first and second bleedings, continuing strong, blood-letting was in this case a third time had recourse to; the patient losing in all upwards of sixty-five ounces of blood within the space of thirty hours, and that with the happiest effects, notwithstanding that the dropsical and other symptoms had existed for nearly two days previously to my seeing him, and that the anasarcous swelling, in the interval between the bleedings, had very considerably increased.

The above observations were chiefly written during the progress of a voyage from the East Indies to England, in the year 1822; since which time I have had an opportunity of perusing Mr. Marshall's Notes on the Medical Topography of the Interior of Ceylon, in the which mention is made of two or three cases of this disease, where the dyspnœa was very troublesome, and for the relief of which blood-letting was had recourse to with immediate advantage. Mr. M.

however adds, that "a more extended clinical experience is still necessary before a due estimate can be made of the effects of the depletory means of cure." I have much pleasure in being able to contribute my mite to the testimony of Mr. Marshall in favor of a mode of treatment, which I have no hesitation in saying, whoever would successfully combat this "hydra disease" would do well to adopt.

In addition to the cases mentioned by Mr. Marshall, I am happy at having it in my power to notice two others, with the history of which I have been recently favored by my friend, Dr. C. Rogers, of Edinburgh, in the treatment of which blood-letting was had recourse to, as in Mr. Marshall's cases, for the purpose of relieving dyspnœa, and was speedily followed by a striking alleviation of all the symptoms. These cases occurred so late as the year 1823.

This evidence in favor of the depletory mode of cure I consider strong, and the more particularly so coming from such authority as that of Dr. Rogers, who for some time practised along with Dr. Christie in the Island of Ceylon, and lately filled the situation of superintending surgeon in the northern division of the Madras settlement; a station where he had ample opportunities of seeing and treating the disease under its every form, and who not long since entertained very different views on the subject to those which he has lately been led to adopt. In allusion to blood-letting in the treatment of this disease, (as found in his Disputatio Inauguralis Medica quædam de Hydrope Asthmatico in Ceylonia, grassante Beriberia, dicto Complectence,) he makes use of the following words:—"Quin et, ad phlebotomiam tandem decursum fuit; vena autem pertusa, morbus cursum ejus funestum celerius absolvit."

### CASE.

November 20th, 1820, ten A.M.—H. C., æt. thirty-two, of a rather spare habit of body, says that he has felt more or less indisposed for the last two days. Complained last night of very acute headache, with a sense of extreme debility and severe dyspnœa, (which last came on him suddenly,) of great numbness, and a disagreeable pricking pain in his thighs and legs, which were much swelled and œdematous; face and abdomen likewise considerably swelled; countenance extremely anxious; complained of feeling very cold; inclined much to vomit; pulse 120, weak and irregular; tongue furred; urine scanty and high coloured; bowels open; skin dry, and cooler than natural. Has lived for the greater part of the last twelve months on the Island of Ceylon.—Was bled to the extent of thirty ounces, which afforded much, and almost immediate, relief; had also four grains of calomel, with ten grains of jalap, given him, for the purpose of acting upon his bowels. This was, however, soon afterwards vomited; when thirty drops of Tinct. Opii were given, with a view to moderate the gastric irritability which existed, and procure sleep.

I visited him this morning at ten A.M. Says that he has passed a very restless night. All the symptoms, though not so urgent as before the bleeding, still continue, particularly the headache and dyspnœa; pulse 116, stronger and rather more regular than at last visit. Blood taken last night of a buffy appearance.

Mittantur mox pleno rivo sanguinis unciæ quinque viginti.—℞. Subm. Hydrargyri ℈j.; Tinct. Opii gtt. iij.; Pulv. Scillæ gr. j. M. statim sum.

Six P.M.—Feels better: dyspnœa and headache much relieved by the bleeding. The Calomel and Laudanum twice repeated since morning, with the addition of six grains of Gamboge to the last dose, for the purpose of acting on his bowels. Has had the fumes of the Hyd. Oxyd. Ciner. twice applied to the surface of the body, which was each time followed by a general glow of heat and free perspiration; and, soon after the first application, completely removed that coldness of the extremities, and indeed of the whole body, from which he appeared to suffer much at the time I first saw him. Œdematous swelling now almost general over the whole surface of the body; pulse ninety-six, softer, and more regular; thirst urgent. Blood taken in the morning slightly buffy.

Omittatur Tinctura Opii.—Repet[r] Hydrarg. Submur. et Pulv. Scillæ tertia quâque horâ.—Suffit. quartâ quâque horâ.—℞. Unguent. Hyd. ℥ij.; Aq. Ammon. ℥j. M. infric. semi uncia de die.—℞. Supertart. Potassæ ℥j. Infunde in aquæ ferventis unciis quindecem per semihoram et cola. Capiat uncias tres pro re nata.

21st.—Has had several copious watery stools since yesterday evening; slept badly; dyspnœa less troublesome; headache better; œdema of extremities slightly diminished; urine still scanty and very high coloured; great apparent debility; thirst incessant; pulse ninety-eight, and pretty full; perspires freely for some time after each fumigation; countenance bloated: tongue furred. Has taken twenty grains of Calomel almost every three hours since yesterday morning; had the fumes of the Hydrargyri Oxydum Cinerium regularly applied to the surface of the body, and the Ung. Hyd. Fort. repeatedly rubbed in during the night, but as yet there is no appearance of ptyalism.

Continuentur medicamenta ut heri præscripta.

Six P.M.—Dyspnœa very much relieved; anasarcous swelling of the abdomen considerably reduced, but the œdema of the lower extremities appears very little diminished; thirst still urgent. Drinks freely of the cream of tartar and water, which keeps his bowels open. Has made upwards of two pounds of urine during the day, not so high coloured as formerly; breath affected by the mercury. Diet, light soup and sago.

Continuentur omnia.

22d.—Spits copiously; slept little during the night, but there is today a most striking alteration for the better: dyspnœa almost entirely gone; anasarcous swelling all over the body very perceptibly diminished; thirst less urgent. Has made nearly six pounds of urine since yesterday afternoon. At times perspires freely; pulse 102; tongue rather loaded; bowels loose. Says he feels much stronger than he has before done since the commencement of the attack; appetite pretty good. Diet, soup and sago.

Omittantur omnia medicamenta.—Utatur Gargarism. ex Aq. font. et Nitrat. Potassæ.

Six P.M.—Complains chiefly of the soreness of his mouth; little or no difficulty of respiration. Quantity of urine since morning, eight pounds. Skin moist; pulse ninety, and pretty strong; no stool during the day. Had a little boiled fowl for dinner.

℞. Potassæ Supertart. ʒvj.; Pulv. Jalap. gr. x. M. fiat pulv. statim sumend.

23d.—Until about four A.M. rested well; since which time he has had frequent watery stools, from the cream of tartar and jalap. Saliva flowing freely; no return of dyspnœa; œdema rapidly diminishing; appetite getting keen; still complains of thirst; pulse eighty-six; urine copious; skin dry. Diet, fowl, soup, and sago.

Contr Garg.—Habeat decoctum hordei pro potu ordinario.—℞. Infus. Gent. comp. ℥ij.; Liquor Ammon. Acetat. ℥ss.; Spirit. Ætheris Nit. ʒij. M. fiat haust. ter in die sumend.—Habeat ter in die Spirit Vin. Holland. unciam ex aq.

Six P.M.—Continues doing well; has had three copious liquid stools since morning. Skin dry and rough; urine, during the last ten hours, upwards of three pounds; pulse ninety-two.

Continuentur medicamenta ut heri, et capiat hora somni.—Opii gr. iv.; Pulv. Ipecac. gr. iv.; Sub. Sulph. Hyd. Flav. gr. ij. M. fiat pil. ij.*

24th.—Slept well, and has perspired very copiously during the night, so much so that it became necessary this morning to remove the blankets which were next him. Œdema daily decreasing; countenance becoming much more natural; mouth continues sore; appetite good; pulse eighty-two.

Continuentur Garg., Decoct. hord., Mist. Diuret., et Sp. Vin. Holland.

Six P.M.—Has had no stool for the last twenty-four hours; skin moist; pulse ninety.

℞. Pulv. Jalap. gr. x.; Supertart. Potassæ ʒiv. M.

25th.—Appears today much better in every respect; has had three copious liquid stools from the purgative. Œdema of the extremities entirely reduced, that of the abdomen so much so that it does not now pit on pressure. Still spits freely; urine of a more natural appearance, and begins to diminish in quantity; ap-

* The above prescription of the late Dr. MARRYAT is perhaps the most effectual diaphoretic that can be given, and at the same time mild in its operation.

petite very keen; thirst much less urgent; skin moist; pulse eighty-two. Diet as before.

**Continuentur medicamenta.**

26th.—Has had a good night. Anasarcous swelling all over the body completely gone. Says that he feels daily getting stronger, but has still considerable numbness of the lower extremities, particularly felt upon attempting to walk; mouth getting better. Has flannel bandages tightly applied from the toes up to the groin on each side, and the parts well rubbed with stimulating liniments.

**Habeat bis in die Vin. Alb. ℥ij.—Continuentur alia ut heri.**

27th.—Continues improving: says his appetite is now so keen that he can scarcely satiate it; general appearance good; skin moist; pulse eighty-six and strong; bowels open. Had a blister applied last night along the course of the spine, in the dorsal region, which has risen well.

**Continuentur medicamenta.**

28th.—Complains only of the soreness of his mouth, which is, however, getting fast well. Walked for nearly a quarter of an hour yesterday, without feeling much fatigued.

**Continuentur medicamenta.**

29th.—Improves in strength daily. No stool for the last twenty-four hours; skin moist; pulse natural.

**℞. Pulv. Jalap. gr. vj.; Supertart. Potassæ ʒij. M. statim sumend.—Contr alia ut heri.**

30th.—Makes little or no complaint today: mouth almost well. Has had two stools from the cream of tartar and jalap given yesterday.

**Contr medicamenta.**

December 1st.—During the last two days has wonderfully improved in appearance. Appetite continues good: pulse regular; skin moist; bowels open; tongue clean; voids about the natural quantity of urine in the twenty-four hours.

**Contr medicamenta.**

2d.—Perfectly well, except a slight numbness of the inferior extremities, which still continues.

**Contr medicamenta.**

3d.—Had a second blister applied last night, about two inches broad, along the whole course of the spine, which has risen well.

**Curetur ulcus Ungt. Sabin.—Contr medicamenta.**

4th.—Continues well in his general health.

**Habeat vini libram indies.—Omittantur alia.**

5th.—Discharged from the sick report, with instructions not to resume his duty until the numbness of the extremities shall have been completely removed.

This individual, in about ten days from the time that he was discharged, had perfectly recovered the use of his limbs, and was in every respect completely cured.

